# Diagnosing and treating Diamond Blackfan anaemia: results of an international clinical consensus conference

**DOI:** 10.1111/j.1365-2141.2008.07269.x

**Published:** 2008-09

**Authors:** Adrianna Vlachos, Sarah Ball, Niklas Dahl, Blanche P Alter, Sujit Sheth, Ugo Ramenghi, Joerg Meerpohl, Stefan Karlsson, Johnson M Liu, Thierry Leblanc, Carole Paley, Elizabeth M Kang, Eva Judmann Leder, Eva Atsidaftos, Akiko Shimamura, Monica Bessler, Bertil Glader, Jeffrey M Lipton

**Affiliations:** 1 The Feinstein Institute for Medical ResearchManhasset, NY; 2 Schneider Children's Hospital, Albert Einstein College of MedicineNew Hyde Park, NY, USA; 3 St. George's Hospital Medical SchoolLondon, UK; 4 Departments of Genetics and Pathology, Uppsala UniversityUppsala, Sweden; 5 Clinical Genetics Branch, Division of Cancer Epidemiology and Genetics, National Cancer Institute, Department of Health and Human ServicesBethesda, MD; 6 Columbia UniversityNew York, NY, USA; 7 Università di TorinoTorino, Italy; 8 Zentrum fuer Kinderheilkunde und Jugendmedizin, Universitaetsklinikum FreiburgFreiburg, Germany; 9 Lund UniversityLund, Sweden; 10 Hôpital Saint-LouisParis, France; 11 NovartisEast Hanover, NJ; 12 National Institute of Allergy and Infectious Diseases, National Institutes of HealthBethesda, MD; 13 Children's HospitalBoston, MA; 14 Washington UniversitySt. Louis, MO; 15 Stanford UniversityPalo Alto, CA, USA

**Keywords:** Diamond Blackfan anaemia, bone marrow failure, cancer predisposition, genetics, treatment

## Abstract

Diamond Blackfan anaemia (DBA) is a rare, genetically and clinically heterogeneous, inherited red cell aplasia. Classical DBA affects about seven per million live births and presents during the first year of life. However, as mutated genes have been discovered in DBA, non-classical cases with less distinct phenotypes are being described in adults as well as children. In caring for these patients it is often difficult to have a clear understanding of the treatment options and their outcomes because of the lack of complete information on the natural history of the disease. The purpose of this document is to review the criteria for diagnosis, evaluate the available treatment options, including corticosteroid and transfusion therapies and stem cell transplantation, and propose a plan for optimizing patient care. Congenital anomalies, mode of inheritance, cancer predisposition, and pregnancy in DBA are also reviewed. Evidence-based conclusions will be made when possible; however, as in many rare diseases, the data are often anecdotal and the recommendations are based upon the best judgment of experienced clinicians. The recommendations regarding the diagnosis and management described in this report are the result of deliberations and discussions at an international consensus conference.

Diamond Blackfan anaemia (DBA; OMIM #205900) was first reported by Josephs (1936) and refined as a distinct clinical entity by Diamond and Blackfan (1938). It is now accepted that the disorder results from a cellular defect in which erythroid progenitors and precursors are highly sensitive to death by apoptosis, leading to erythropoietic failure (Lipton *et al*, 1986; Tsai *et al*, 1989; Perdahl *et al*, 1994; Ohene-Abuakwa *et al*, 2005; Miyake *et al*, 2008). DBA is a member of a rare group of genetic disorders characterized by pro-apoptotic hematopoiesis leading to bone marrow failure, congenital anomalies (Gripp *et al*, 2001) and predisposition to cancer (Lipton *et al*, 2001), known as the inherited bone marrow failure syndromes (IBMFS) (Young & Alter, 1994).

The first DBA gene, mutated in approximately 25% of patients, has been cloned and was identified as *RPS19*, which codes for a ribosomal protein located at chromosome 19q13.2 (Gustavsson *et al*, 1997; Draptchinskaia *et al*, 1999). The function of this protein in ribosome biogenesis is poorly understood. In patients with an *RPS19* mutation, it has been proposed that the disease results from rps19 protein haploinsufficiency, i.e. where the protein produced by a single copy of a normal gene is not sufficient to produce normal function (reviewed in Gazda *et al*, 2004). Studies have demonstrated the repair of defective hematopoiesis by increased rps19 protein expression in both rps19-deficient patient-derived progenitors (Hamaguchi *et al*, 2002) and in rps19 knockdown cellular models (Celiker *et al*, 2004; Ebert *et al*, 2005; Flygare *et al*, 2005). Recently *de novo* mutations have been identified in ribosomal proteins rps24, encoded by *RPS24* at chromosome 10q22-q23 (Gazda *et al*, 2006) and in rps17, encoded by *RPS17* at chromosome 15q25.2 (Cmejla *et al*, 2007), each in approximately 2% of patients. Furthermore mutations in large ribosomal subunit-associated proteins rpl5, rpl11, and rpl35a, have been described in 10%, 6·5% and 2% of patients (Farrar *et al*, 2007; Gazda *et al*, 2007). To date, approximately 50% of DBA patients have a single mutation in a gene encoding a ribosomal protein. These findings implicate DBA as a disorder of ribosome biogenesis and/or function. Indeed a recent study demonstrates that *RPS19* is essential for maturation of the 40S ribosomal subunit (Flygare *et al*, 2007). The relationship of ribosomal protein haploinsufficiency to faulty ribosome biogenesis has been recently reviewed (Ellis & Lipton, 2007; Flygare & Karlsson, 2007).

Scientific advances in DBA and the availability of reliable clinical data from well-characterized patient populations in international registries have resulted in a reconsideration of the diagnostic criteria and clinical management of DBA, developed at the 6th Annual Diamond Blackfan Anemia International Consensus Conference held in New York on April 16–18, 2005 and delineated in this “consensus document”.

## Establishing the diagnosis

Since the original description of the disorder (Diamond & Blackfan, 1938) and the landmark review in 1976 (Diamond *et al*, 1976), a number of developments have both clarified and complicated the designation of patients to the diagnosis of DBA. In patients who have unambiguous red cell failure defined by macrocytic anaemia and reticulocytopenia and decreased or absent red cell precursors in the bone marrow, the differential diagnosis of pure red cell aplasia includes DBA and a diverse array of acquired disorders (Table I). In children, in particular, transient erythroblastopenia of childhood (TEC) should be the major consideration (Table II). Pearson syndrome, parvovirus B19, human immunodeficiency virus (HIV) and other infections, drugs and toxins as well as immune-mediated disease should be ruled out before the diagnosis of DBA can be established. The presence of parvovirus genome in the bone marrow should be assessed by polymerase chain reaction (Parekh *et al*, 2005). The diagnosis of thymoma should be considered in adults, as it rarely occurs in children. DBA is diagnosed in adults more often than previously thought (Balaban *et al*, 1985) although other causes of red cell failure are more common. Finally, the other IBMFS, in particular Shwachman Diamond syndrome and Fanconi anaemia, should be considered, as macrocytic anaemia may also be a frequent hematological manifestation in these syndromes.

**Table I:** Classification of pure red cell aplasia*.

Inherited
Diamond Blackfan anaemia
Acquired PRCA
Primary
Autoimmune [includes Transient Erythroblastopenia ofChildhood (TEC)]
Pearson syndrome (sideroblastic anaemia with vacuolatederythroid precursors)
Preleukemic
Idiopathic
Secondary, associated with:
Thymoma
Hematological malignancies
Chronic lymphocytic leukemia
Large granular lymphocyte leukemia
Chronic myelocytic leukemia
Acute lymphoblastic leukemia
Hodgkin lymphoma
Non-Hodgkin lymphomas
Multiple myeloma
Waldenström macroglobulinemia
Myelofibrosis with myeloid metaplasia
Essential thrombocythemia
Solid Tumours
Carcinoma of the stomach
Adenocarcinoma of the breast
Adenocarcinoma of bile duct
Squamous cell carcinoma of the lung
Epidermoid carcinoma of the skin
Carcinoma of the thyroid
Renal cell carcinoma
Carcinoma of unknown primary site
Kaposi sarcoma
Infections
Human B19 parvovirus
Human immunodeficiency virus (HIV)
T-cell leukemia-lymphoma virus
Epstein-Barr virus (infectious mononucleosis)
Viral hepatitis
Mumps
Cytomegalovirus
Atypical pneumonia
Meningococcemia
Staphylococcemia
Leishmaniasis
Chronic haemolytic anaemias
Collagen vascular and autoimmune diseases
Systemic lupus erythematosus
Rheumatoid arthritis
Mixed connective tissue disease
Sjögren syndrome
Autoimmune multiple endocrine gland insufficiency
Autoimmune hypothyroidism
Autoimmune chronic hepatitis
Drugs and chemicals
Pregnancy
Severe renal failure
Severe nutritional deficiencies (rehabilitation of kwashiorkor)
Miscellaneous
Post-ABO incompatible bone marrow transplantation
Angioimmunoblastic lymphadenopathy
Anti-erythropoietin antibodies (spontaneous or post-treatmentwith erythropoietin)

*Modified with permission from Dessypris and Lipton, 2003.

**Table II:** Differential diagnosis of DBA *versus* TEC.*

	Diamond Blackfan anaemia (DBA)	Transient erythroblastopenia of childhood (TEC)
Pure red cell aplasia	Present	Present
Age	Younger than 1 year	Older than 1 year
Inheritance	Sporadic and dominant inheritance	Not inherited
Congenital anomalies	Present	Absent
Mean corpuscular volume (MCV)	Elevated	Normal
HbF	Elevated	Normal
i RBC antigen	Present	Absent
Erythrocyte ADA (eADA) activity	Elevated	Normal

All RBC characteristics except eADA activity are helpful only when tested in a reticulocytopenic child. During recovery from TEC, a transient wave of fetal-like erythropoiesis with elevated MCV, HbF, and i RBC antigen may be detected. This testing is valid in untransfused children only.

*Modified with permission from Dessypris and Lipton, 2003.

Based on data from Link and Alter, 1981.

Diamond Blackfan anaemia was first described as a disorder of impaired red cell production in children. The presentation in adults, the risk of other cytopenias, the predisposition to cancer and the high incidence of birth defects are now being quantified (Willig *et al*, 1999a; Vlachos *et al*, 2001a; Campagnoli *et al*, 2004; Ohga *et al*, 2004; Orfali *et al*, 2004). The diagnostic criteria for DBA, presented in 1976 (Diamond *et al*, 1976), have remained the accepted standard. These include anaemia, presenting prior to the first birthday, with near normal, but variable, neutrophil and/or platelet counts, reticulocytopenia, macrocytosis and normal marrow cellularity with a paucity of red cell precursors. Newborns with DBA rarely present with hydrops fetalis (Rogers *et al*, 1997; Dunbar *et al*, 2003). Certainly these diagnostic criteria define “classical” DBA. It is apparent from the study of multiplex families that affected individuals may present in “non-classical” ways. For example, individuals may present at an age greater than one year, only with congenital anomalies, without anaemia or with a mild hematological phenotype (macrocytosis only). These cases of “non-classical” DBA need to be more carefully identified, particularly when reproductive choices and transplant donor decisions are being made. Furthermore, as the risk of malignancy and other complications of DBA are better defined, the necessity of making a diagnosis in “asymptomatic” individuals will become more important.

Diagnostic and supporting criteria for the diagnosis of DBA are described in Table III. These criteria are subject to future modification as more evidence-based data are accumulated. A diagnosis of “classical” DBA is made if all the diagnostic criteria are met. When there is a positive family history, an otherwise normal individual should be considered as having “non-classical” DBA if a mutation shared by affected family members is present. Anyone suspected of having DBA, but with insufficient diagnostic criteria, should be considered as having sporadic, non-classical DBA if a reported mutation is present. A patient can be assigned as having a “probable” diagnosis, with a decreasing degree of certitude if; three diagnostic criteria are present along with a positive family history; two diagnostic criteria and three minor supporting criteria are present; or, a positive family history and three minor supporting criteria are evident, even in the absence of diagnostic criteria. Of note, macrocytosis may be masked by iron deficiency or thalassemia minor and, in the newborn, can be obscured by residual fetal erythrocytes. The erythrocyte adenosine deaminase (eADA) activity, not influenced by prior transfusions, is elevated (≥3 SD) in 80–85% of patients classified as having DBA (Glader & Backer, 1988). In contrast, 90% of patients classified as having TEC have normal eADA activity. Elevated eADA activity, increased fetal hemoglobin (HbF) and mean corpuscular volume (MCV) are not very strong independent criteria, however, these factors should be seriously considered when evaluating a sibling as a stem cell transplant donor. Steroid responsiveness is not considered a diagnostic criterion and corticosteroids should not be administered until a diagnosis is made. If the first three diagnostic criteria are present, but there is no paucity of red cell precursors in the bone marrow and no supporting criteria, the diagnosis of DBA cannot be made. A bone marrow evaluation should be repeated at a later date as red cell marrow hypoplasia may develop after anaemia and reticulocytopenia. Furthermore, thrombocytopenia and neutropenia are not uncommon findings (Giri *et al*, 2000). Thus the presence of additional cytopenias does not preclude the diagnosis of DBA in a patient with red cell aplasia and may be severe enough to require treatment.

**Table III:** Diagnosing Diamond Blackfan anaemia (DBA).

Diagnostic criteria
Age less than 1 year
Macrocytic anaemia with no other significant cytopenias
Reticulocytopenia
Normal marrow cellularity with a paucity of erythroid precursors
Supporting criteria
Major
Gene mutation described in “classical” DBA
Positive family history
Minor
Elevated erythrocyte adenosine deaminase activity
Congenital anomalies described in “classical” DBA
Elevated HbF
No evidence of another inherited bone marrow failure syndrome

An evaluation of the family of a proband is necessary. All immediate family members should be evaluated with a thorough relevant history (anaemia, cancer, birth defects, etc.), complete blood count including red cell indices, eADA activity and HbF. If the proband has a mutation, then the parents and siblings need to have appropriate mutation analysis. The nature of any other positive findings will dictate the extent of the family evaluation.

## Congenital anomalies

Congenital anomalies mainly involve the head, upper limbs, heart and genitourinary system (Table IV) (Ball *et al*, 1996; Janov *et al*, 1996; Ramenghi *et al*, 1999; Willig *et al*, 1999a; Gripp *et al*, 2001; Vlachos *et al*, 2001a). In a study of 80 DBA patients from a 20-year birth cohort in the United Kingdom (Ball *et al*, 1996), 35% of classical DBA patients were found to have one or more unequivocal congenital anomalies, not including growth retardation. A similar proportion has been described in the French, Italian, and North American registries (40%, 46%, and 47%, respectively) (Ramenghi *et al*, 1999; Willig *et al*, 1999a; Lipton *et al*, 2006). More than one anomaly is found in up to 25% of individuals. There is a wide range of severity of congenital anomalies in DBA, with variability even within the same kindred.

**Table IV:** Range of congenital anomalies observed in Diamond Blackfan anaemia (DBA).

Craniofacial	Hypertelorism
	Broad, flat nasal bridge
	Cleft palate
	High arched palate
	Microcephaly
	Micrognathia
	Microtia
	Low set ears
	Low hair line
	Epicanthus
	Ptosis
Ophthalmological	Congenital glaucoma
	Strabismus
	Congenital cataract
Neck	Short neck
	Webbed neck
	Sprengel deformity
	Klippel-Feil deformity
Thumbs	Triphalyngeal
	Duplex or bifid
	Hypoplastic
	Flat thenar eminence
	Absent radial artery
Urogenital	Absent kidney
	Horseshoe kidney
	Hypospadias
Cardiac	Ventricular septal defect
	Atrial septal defect
	Coarctation of the aorta
	Complex cardiac anomalies
Other musculoskeletal	Growth retardation
	Syndactyly
Neuromotor	Learning difficulties

The list includes the anomalies that are most characteristic of DBA, but is not exhaustive. Multiple anomalies, most commonly including craniofacial, are present in up to 25% of affected individuals.

Craniofacial anomalies are the most common, representing 50% of congenital anomalies reported to the North American DBA Registry, with hypertelorism and broad flat nasal bridge contributing to the classic DBA facies described by Cathie (1950). Thumb anomalies have been described in 9–19% of patients. The severity of abnormalities ranges from hypoplasia of the thenar eminence to absence of the radius or forearm, duplicated, bifid or the classic triphalangeal thumb (Aase syndrome) (Aase & Smith, 1969). Renal and cardiac anomalies have each been described in up to 7% of patients in the UK, France and Italy. However, in the North American Diamond Blackfan Anemia Registry (DBAR), where the majority of patients undergo formal genitourinary and cardiac imaging, the prevalence is higher at 19% and 15%, respectively.

A low birth weight is reported in approximately 25% of patients (Ball *et al*, 1996; Willig *et al*, 1999a). Growth retardation (height below 3rd centile for age) is described in approximately 30% of children. It must be noted that stature is difficult to evaluate in the context of severe anaemia, iron overload and chronic corticosteroid use. Children on chronic steroid therapy, while over-represented in the bottom quartile in the UK Registry, did not predominate in the group with height less than the 3rd centile. Growth retardation is commonly associated with physical abnormalities, and can thus be considered part of the spectrum of congenital anomalies. However, as final height is influenced by other factors, the true prevalence of constitutional short stature is not accurately known (Chen *et al*, 2005).

Renal imaging and echocardiogram are recommended, with nephrology, urology and cardiology referral as appropriate. Other specialist referrals should also be made as indicated. The growth of children with DBA should be carefully monitored, especially during adolescence, and prompt referral should be made for children with impaired growth velocity. The judicious use of corticosteroids, maintenance of an adequate hemoglobin level and appropriate iron chelation are essential in optimizing growth. The occurrence of equivalent anomalies in otherwise asymptomatic siblings and parents of DBA patients is likely to reflect non-classical DBA, and should prompt further investigation.

## Genetics and reproductive choices

Recent data showed that approximately 40–45% of DBA cases are familial with autosomal dominant inheritance (Orfali *et al*, 2004), the remainder being sporadic or familial with seemingly different patterns of inheritance. The transmission is often unpredictable with examples of reduced penetrance and co-existence of both mild and severe forms within a pedigree (variable expressivity) (Gustavsson *et al*, 1997; Willig *et al*, 1999a,b; Gripp *et al*, 2001). Indeed up to 30% of these families were identified from family studies on “sporadic” patients (Orfali *et al*, 2004). Genetic counseling is essential in DBA because of the risk of recurrence. The incidence of DBA is estimated to be between 1/100 000 and 1/200 000 without ethnic predilection, with both sexes equally affected (Ball *et al*, 1996; Ramenghi *et al*, 1999; Willig *et al*, 1999a; Ohga *et al*, 2004; Lipton *et al*, 2006). Routine cytogenetic analysis is usually normal. The genetic basis of DBA is heterogeneous and approximately 50% of patients are heterozygous for *RPS17, RPS19, RPS24, RPL5, RPL11, or RPL35A*. All the mutations to date have been found in one allele, resulting in severe loss of function or protein haploinsufficiency. Homozygosity, lethal in the mouse knockout, and compound heterozygosity have not been described in DBA patients.

The most common mutation is found in *RPS19* thus far. A recent review (Lipton, 2007) describes 113 different *RPS19* mutations associated with DBA (Draptchinskaia *et al*, 1999; Matsson *et al*, 1999; Willig *et al*, 1999b; Cmejla *et al*, 2000; Ramenghi *et al*, 2000; Proust *et al*, 2003; Campagnoli *et al*, 2004; Gazda *et al*, 2004; Orfali *et al*, 2004). There is no clear correlation between the type of *RPS19* mutation and the degree of hematological manifestation. Identical mutations may be associated with a wide range of clinical presentations, even within a family; thus, even though dominantly inherited, their expression is clearly modified by other factors.

Approximately 10–15% of cases are from multiplex families with classical DBA evident in more than one family member. Approximately two-thirds of these familial cases appear autosomal dominant whereas one-third could be consistent with autosomal recessive inheritance (Willig *et al*, 1999b; Campagnoli *et al*, 2004; Gazda *et al*, 2004, 2006; Orfali *et al*, 2004). However “true” recessive forms of DBA have not yet been ascertained and a dominant inheritance with a reduced penetrance, or rarely gonadal mosaicism (Cmejla *et al*, 2000), is a likely alternative. Identifying family members who are likely to have silent forms of the disease is important in order to provide reproductive counseling and to exclude them as stem cell transplant donors (Orfali *et al*, 1999).

In a family in which classical DBA is present in the parent and offspring, or in two or more siblings, the recurrence risk is up to 50%. In a presumed sporadic case, the recurrence risk depends on the presence of a DBA-associated mutation or any manifestations in first-degree relatives. A reported DBA mutation in two successive generations, regardless of clinical presentation, results in a 50%*a priori* risk for a second affected child. If the mutation of the proband is excluded in both parents, this is likely to be a new mutation with a recurrence risk related to the possibility of gonadal mosaicism (Cmejla *et al*, 2000) or non-paternity. In a few reported cases a partial or complete deletion of *RPS19* has been identified. Analysis of a deleted gene by DNA sequencing may result in a “false” normal sequence obtained from the intact allele only. If no mutation is identified in the proband and if elevated eADA activity, Hb F and/or MCV are found in asymptomatic first-degree relatives, the recurrence risk should be stated as 50% (Orfali *et al*, 2004). However the possibility of false positive elevation in these parameters may reduce the risk to 30–40%. If the analysis of eADA activity, Hb F and MCV is normal, the recurrence risk is estimated to be 5–10%, due to the possibility of false negatives.

Prenatal diagnosis is possible for DBA if a mutation is identified in the family. More recently, preimplantation genetic diagnosis (PGD) has become an option to greatly reduce the risk of a second affected child. This can be performed in families with a mutation found in a parent, in order to select and implant embryos without risk for DBA. This method can also be combined with PGD for human leucocyte antigen (HLA) typing for families with an affected child in need of an HLA-matched stem cell transplant. If mutation analysis (50% probability) and HLA typing (25% probability) are combined, the maximal success rate is only 12·5% of embryos. PGD for HLA typing for families at risk for DBA but without a mutation has also been performed with success (Verlinsky *et al*, 2004; Kuliev *et al*, 2005). This option needs to be combined with clinical and laboratory investigation of family members followed by information to the couple about their specific recurrence risk for DBA as described above. The practical and ethical considerations of PGD for HLA typing alone in DBA, when a genetic diagnosis is unavailable and when no, or only equivocal, risk factors are identified, are being debated. Furthermore, this method is not universally available (Dobson, 2003; Dyer, 2004, 2006; Grewal *et al*, 2004).

## Cancer predisposition

Until recently, information with regard to the cancer risk in patients with DBA has been limited primarily to case reports: 29 cases (as well as three with myelodysplasia who did not develop acute myeloid leukemia) were reported among more than 700 patients in the literature (updated from Alter, 2003; Yaris *et al*, 2006). The reported proportion of 4% is higher than the expected <1% for a cohort less than 30 years of age. The median age for cancer in these reports was 15 years (range 1 to 43 years), much younger than the median of 68 years in the general population (Ries *et al*, 2001). The cases of cancer in DBA patients may reflect an over-reporting bias and thus the true risk is unknown. Better information may be derived from longitudinal follow-up of cohorts of DBA patients. The crude frequency reported from Boston Children's Hospital was 5/76 or 6·6% (Janov *et al*, 1996), the French series 9/240 or 3·8% (Willig *et al*, 1999a), the DBAR 8/420 or 1·9% (Lipton *et al*, 2001), the Italian registry 0/96 (unpublished observations), and the National Cancer Institutes IBMFS cohort 3/58 or 5% (unpublished observations), compared to an expected 0·5% after adjustment for age, sex, and birth cohort. None of these data have yet been analyzed adequately using time-dependent and appropriately adjusted methods.

While the published data are insufficient to define the risk of cancer in DBA, the types of malignancy reported are informative. The literature includes 11 cases of acute leukemia (10 acute myeloid leukemia [AML] and one acute lymphoblastic leukemia), three cases of myelodysplastic syndrome that did not develop AML, six cases of sarcoma (five osteogenic, one soft tissue), three Hodgkin lymphoma, two breast cancer, two hepatocellular carcinoma, and one each of melanoma, fibrohistiocytoma, non-Hodgkin lymphoma, gastric cancer and colon cancer.

The role of any DBA genes in this process is unknown. The mechanism for the increased incidence of AML may be related to selective pressure in patients with pro-apoptotic states, as described in the IBMFS (Lensch *et al*, 1999). It is unknown whether there is any genotype-phenotype correlation between *RPS19* mutations, or other DBA genes, and cancer predisposition. Therefore screening cannot be targeted to a specific genotype. It is important to note that DBA is unequivocally one of the IBMFS with a predisposition to malignancy.

We may speculate that management of cancer with chemotherapy may be compromised, because of the reduced bone marrow reserve seen in patients with a hematopoietic progenitor defect (Giri *et al*, 2000), which could result in delayed recovery from marrow suppression and increased toxicity. There may be a role for early hematopoietic stem cell transplantation (HSCT) in some patients. The survival of DBA patients with cancer appears to be less than that of patients with similar cancers in the general population, but this cannot be analyzed statistically using case reports in the literature. A prospective cohort is required for this purpose (Lipton *et al*, 2006).

Typically a periodic history and physical examination with blood count monitoring is done at 4–6 month intervals in stable DBA patients. If abnormalities appear in the blood, bone marrow aspirate, biopsy, and cytogenetic studies (karyotype, as well as FISH analysis for abnormalities in chromosomes 5, 7, and 8) should be performed. An annual bone marrow examination to determine early signs of marrow evolution to myelodysplasia or AML, done by some centres, is not considered standard of care. It may be prudent to minimize the radiation exposure from diagnostic tests, given the increased risk of sarcoma. Nevertheless, there should be a low threshold for investigation of bone or joint pain in DBA patients.

## Corticosteroid therapy

Corticosteroids remain the mainstay of treatment in DBA more than half a century after the original report of their efficacy (Gasser, 1951). Their mechanism of action in DBA is still unknown and under investigation. Approximately 80% of DBA patients respond to an initial course of steroids. There is currently no reliable way to predict steroid responsiveness. The *RPS19* mutation status has not been predictive of response in any series.

Once steroid therapy is started, an increase in hemoglobin is usually seen within two to four weeks. The dose is then tapered to determine the minimum dosage required for continued transfusion independence. The maintenance dose in steroid responders is highly variable with some individuals requiring extremely small doses. The mechanism of this variable response is unknown. In contrast, some patients become refractory to steroids despite an initial response. In others, steroid therapy may have to be discontinued due to unacceptable side effects. In over 20% of DBA patients, steroids (or red cell transfusions) may eventually be stopped completely with continued maintenance of adequate hemoglobin levels, a so-called “remission” or more appropriately, treatment independence.

There is limited long-term follow-up data on the use of steroids in infancy. Premature infants receiving steroid therapy have a decrease in growth velocity (Stark *et al*, 2001), continued growth delay and neuromotor dysfunction in toddlerhood (Yeh *et al*, 1998) and delayed motor milestones. Thus in babies with DBA, the need for relatively high doses and prolonged duration of steroid therapy should be balanced against the possible detrimental effects. Learning difficulties were rarely reported in the UK registry but were more common in patients with large deletions, possibly consistent with a contiguous gene syndrome (Orfali *et al*, 2004).

Based upon these observations and the lack of evidence that a delay in starting a trial of steroids affects responsiveness, steroid therapy is not generally recommended in babies under 6 months of age. Some consensus participants recommend a steroid trial beginning at one year of age, but there is no general consensus. An earlier trial of steroids may be considered if there is poor venous access, or where the safety of the blood supply is questionable. When steroids are started in babies less than 1 year of age, growth and neuromotor development should be closely monitored. Prednisone, or prednisolone, is most commonly used, although the steroid preparation does not appear to influence response. The recommended starting dose is 2 mg/kg of prednisone, or the glucocorticoid equivalent, given as a single daily dose in the morning. Some clinicians use divided doses. Steroids should be started once the post-transfusion hemoglobin is at a level of 80–90 g/l, low enough to prevent erythroid suppression, but high enough to allow time for response before the next transfusion. This starting dose should be given for a maximum of 4 weeks. If transfusion independence is not achieved after this time, the steroids should be discontinued. Continuation of steroids or dose escalation in non-responders beyond this time is unwarranted.

There are little data to support a specific steroid tapering schedule. Based on consensus, once the patient responds, the steroids should be weaned to an average daily dose of 1 mg/kg per day over 8–12 weeks. Reducing to an alternate day dosing schedule, often increasing the first day's dose while decreasing the second day's dose, can accomplish this. The dose should then be further tapered slowly to find the minimal maintenance dose necessary to keep the hemoglobin in the range of 80–100 g/l, the target level being determined by the individual patient's requirement to sustain growth and activity. The recommended maximum maintenance dose is ≤1 mg/kg every other day or ≤0·5 mg/kg daily. Steroids should be tapered more slowly, and with great care, below this level to reduce the risk of overshooting the minimal effective dose for that individual. If this occurs, the dose should be immediately increased to the previous level at which the hemoglobin was within target range. The hemoglobin should be monitored closely during this phase because a too rapid taper often necessitates the reinstitution of the initial 2 mg/kg per day dose to re-achieve response. If an acceptable hemoglobin cannot be sustained at the recommended dose, steroids should be tapered and discontinued. Some patients require very small doses for continued response.

The steroid dose in partial responders (patients with decreased transfusion requirement on steroids) should be tapered to find the minimal effective dose like complete responders. If there is no reduction in transfusion requirement at this level, steroids should be tapered and discontinued. Some experts do not favor the use of both modalities simultaneously and discontinue steroids if transfusion independence cannot be achieved.

An increase in the maintenance dose of steroids is usually not required for transient falls in hemoglobin, as may be seen with non-specific viral infections. Occasional transfusions may be given. As children grow they should be permitted to outgrow their dose with upward dose adjustments only if the steady state hemoglobin decreases significantly. Parvovirus B19 infection should be considered as a possible cause of an acute, prolonged drop in hemoglobin (Tchernia *et al*, 1993), although patients have sustained this infection with no effect. A proportion of steroid responders become increasingly resistant, requiring progressively higher doses to maintain their hemoglobin in the target range. Girls, in particular, may show a reduction in steroid sensitivity at puberty. It is important to be careful not to escalate the steroid dose above the recommended limit. Transfusion therapy may be necessary during this time. The combined oral contraceptives and other estrogen mimetic agents should be avoided in steroid-dependent women, as oestrogens may induce steroid refractoriness. Those patients who are treatment independent may have their stability disrupted with the introduction of estrogen derivatives. A repeat trial of steroids may be considered for non-responders, as response to a second course has been described anecdotally.

### Side effects and monitoring

The onset of anaemia during early infancy and the prolonged duration of steroid therapy make DBA unique with respect to the risk of side effects (Table V). A review of all possible steroid side effects is beyond the scope of this article, however those of specific interest to DBA patients are highlighted. The importance of steroid side effects in DBA is illustrated by data from the French registry, in which complications due to steroid therapy, including hypertension, diabetes mellitus, and growth retardation, were noted in 20% of patients on long-term steroid therapy (Willig *et al*, 1999a). In North America, where steroid dosing and monitoring practices may vary, the DBAR has documented 22% of patients on steroids with pathological fractures and 12% with cataracts (Lipton *et al*, 2006). Avascular necrosis, especially affecting the femoral head, is a potential risk and should be investigated in patients with joint pain and stiffness.

**Table V:** Summary of steroid side effects.

Cosmetic	Hirsuitism, moon face, facial erythema,striaie, acne, weight gain
Behavioral	Hyperactivity, depression, psychosis
Endocrine	Adrenal suppression, impaired glucosetolerance, diabetes mellitus,menstrual irregularities
Fluid and electrolytes	Hypertension, hypokalemia, hypocalcemia
Skeletal	Osteopenia, avascular necrosis, fractures
Growth	Impaired growth velocity, especiallyat puberty
Muscular	Myopathy affecting proximal muscles
Immunosuppression	Varicella, *Pneumocystis* pneumonia, candida
Ophthalmological	Cataract
Neurological	Pseudotumor cerebri
Gastrointestinal	Gastritis, perforation, pancreatitis
Cardiovascular	Hypertension

Deaths of DBA patients, especially infants, on steroid therapy have been attributed to *Pneumocystis jiroveci* (formerly *carinii*) pneumonia (Huh *et al*, 2002). Therefore, *Pneumocystis* pneumonia prophylaxis is indicated for patients receiving steroids at a dose of >2 mg/kg, for those undergoing prolonged treatment with >1 mg/kg and for those receiving any additional immunosuppressive therapy. Sulfamethoxazole-trimethoprim is the prophylactic treatment of choice at 5 mg/kg per day in two divided doses per day, on three consecutive days per week. Alternative treatment with Pentamidine, Dapsone or Atavoquone can be used for sulfa allergic patients or in patients with concurrent or intermittent neutropenia.

The initiation of high-dose steroid therapy in infancy, before the acquisition of immunity to live vaccines, also carries an additional potential risk. A prednisone (or equivalent) dose of <2 mg/kg per day or <20 mg/d for body weight greater than 10 kg is applied by the American Academy of Pediatrics as the cut-off limit for the safe administration of live vaccines (American Academy of Pediatrics, 2006). Varicella can be fatal in non-immune individuals on steroid therapy. Varicella immunization is recommended for non-immune individuals older than 12 months of age (Krause & Klinman, 1995). Non-immune individuals being treated with steroids should be advised to seek urgent medical attention following exposure to chickenpox. A hepatitis B immunization program is also recommended.

United Kingdom guidelines on the management of bone density in adults on steroid therapy recommend dual energy X-ray absorptiometry (DEXA) scanning for patients receiving >7·5 mg/d for more than 6 months, and repeated every 1–3 years (Eastell *et al*, 1998) with the addition of bisphosphonate therapy for those patients on >15 mg/d for more than 6 months. Bisphosphonate therapy has been used in a limited number of children with secondary osteoporosis from corticosteroid administration. Although short-term use has been well tolerated, there is insufficient evidence to support the use of bisphosphonates as standard therapy (Ward *et al*, 2007).

Growth retardation is a very important complication of steroid therapy in a disorder already associated with short stature. Growth curves should be closely monitored especially during periods of high growth velocity, such as in infancy and puberty. Steroid dosage should be kept to a minimum during these times. A growth plateau may indicate that steroids should be interrupted and substituted by a short-term transfusion program. Growth hormone therapy has been used successfully to increase growth velocity (Scott *et al*, 2004), but early, prolonged exposure to pharmacological doses of growth hormone should only be carefully considered on a case-by-case basis in DBA. As there is an increased risk of malignancy in DBA, including osteogenic sarcoma, the use of growth hormone should be individualized, although no relationship between the administration of growth hormone and cancer has been established (Alter, 2004).

Patients should be made aware of the danger of stopping steroids suddenly and of the need to inform any medical practitioner of current and past steroid treatment in order that stress doses be administered under appropriate conditions, i.e. surgery, sepsis, injury, and other emergencies (Walsh & Dayan, 2000). Maintenance treatment with steroids should be given as an early morning dose to minimize adrenal suppression. An endocrinological evaluation may be helpful in guiding a steroid taper after prolonged use.

## Transfusion therapy

After an initial period of transfusion at diagnosis, chronic transfusion therapy with packed red blood cells is begun once it has been established that the patient is not responsive to corticosteroids. Since suppression of erythropoiesis is not a goal of transfusion in DBA, as it is in thalassemia major, trough hemoglobin levels of 80 g/l are usually acceptable for maintaining adequate growth and development. No data are available on the incidence or prevalence of alloimmunization in DBA patients, thus, extended panel crossmatching should be performed according to institutional policies. Red blood cell units should be leucocyte depleted.

### Assessment of iron overload

Iron is released from the breakdown of red blood cells by macrophages in the reticuloendothelial system. Since iron is not recycled into new red cells in DBA and there is no specific mechanism for excretion, iron accumulates in tissues. Eventually, as the capacity for safe sequestration of the excess iron is surpassed, extensive iron-induced injury develops in the heart, liver, pancreas, thyroid, and other organs. With transfusional iron overload, the onset of toxic manifestations, the pattern of organ involvement and the severity of tissue damage are known to be influenced by a variety of factors, including the magnitude of the body iron burden, the rate of iron loading, the distribution of excess iron between the reticuloendothelial storage compartment and the parenchymal cells of sensitive tissues, and the amounts of non-transferrin-bound iron in the plasma (Brittenham, 1995). Data on the rate of iron accumulation and its effect on different tissues are available in transfusion-dependent thalassemia patients (Olivieri & Brittenham, 1997), but not in DBA, where ineffective erythropoiesis is not a concern. In a 10-year prospective study of the quantitative relationship between the risk of death from cardiac disease and the magnetically determined hepatic iron concentration, a threshold was identified for deaths from cardiac causes equivalent to about 80 μmol iron/g liver, wet weight (15 mg iron/g liver, dry weight) (Brittenham *et al*, 1994). From these studies, an optimal range of liver iron of 3–7mg/g (dry weight) is suggested for regularly transfused and chelated thalassemia patients. Though it is possible that patients with DBA may tolerate higher levels, there are no data to support this hypothesis. These constraints must be considered when adopting these recommendations.

Without chelation, the hepatic iron concentration in patients receiving monthly transfusions would be predicted to rise from the ideal range (3–7 mg/g, dry weight) to the high-risk range (>15 mg/g, dry weight) in just 12 months. Effectively chelating a patient to prevent tissue deposition and morbidity requires accurately assessing the body's iron load on a regular basis. The hepatic iron concentration is the most reliable measure of iron burden (Angelucci *et al*, 1995, 2000). The “gold standard” for estimating hepatic iron concentration is measurement of non-haem iron in a liver tissue biopsy sample. Non-invasive and validated methods of measuring liver iron include magnetic susceptometry (SQUID) (Brittenham *et al*, 1982, 1989) and magnetic resonance imaging (MRI) (Gandon *et al*, 2004; Voskaridou *et al*, 2004; St Pierre *et al*, 2005). SQUID has the disadvantages of limited accessibility (only two instruments in the United States and two in Europe) and the inability to study iron deposition in the other organs. MRI is more readily accessible and can image the heart, pancreas and pituitary gland, but testing requires specific sequences, and measurements in the heart are still investigational. An assessment of liver iron concentration is recommended every 12–18 months (more frequently if the patient is in the high risk range and intensive chelation is underway to reduce it more rapidly). Of note, one-third of transfusion-dependent DBA patients in an Italian study had significant iron overload as determined by SQUID imaging (Ramenghi U, Piga A, personal communication). Though several groups have shown that measuring the serum ferritin level is not a reliable indicator of the body iron load, in the absence of the availability of SQUID, MRI or liver biopsy data, it continues to be used to monitor the iron load.

Many methods have been proposed to assess cardiac iron (Hershko *et al*, 2004), including T2* (Anderson *et al*, 2001), R2 (Wood *et al*, 2004) and signal intensity ratio (SIR) (Jensen *et al*, 2003) measurements. T2* is being used clinically, with a value of <8 ms considered an indicator of high risk for cardiac disease. However, it does not have a high positive predictive value, and should be interpreted with caution. Annual monitoring of cardiac function by echocardiography or MRI, and Holter monitoring has some utility and is recommended, even though the onset of cardiac failure is often sudden and may occur even after reassuring normal parameters on routine echocardiography and electrocardiography. Detailed endocrine evaluation, including bone densitometry, is recommended at intervals based on age, compliance with chelation therapy and iron status.

### Chelation therapy

There are few data to guide the initiation of chelation therapy. However chelation should be initiated when the hepatic iron concentration has reached 6–7 mg/g, dry weight, which corresponds to approximately 170–200 ml/kg of transfused packed red cells. If SQUID and MRI are not readily available at this time, a liver biopsy should be performed although it may be deemed too invasive or unavailable for some patients. In such situations, a ferritin level of 1000–1500 μg/l has been used as a starting point and, although not perfect, may be used to monitor iron balance. Initiating iron chelation too early may result in toxicity from the chelator, whereas delaying it risks tissue deposition and consequent organ dysfunction and morbidity. Standard chelation, until recently, has been limited to the use of deferoxamine (Desferal®, Novartis) (Olivieri & Brittenham, 1997), initiated at a dose of 40 mg/kg by subcutaneous infusion over 8–12 h, 4–6 nights per week. This may be modified using the hepatic iron concentration as a guide (or, when necessary, serum ferritin level); if the level is low, chelation may be reduced to 3–4 nights per week, or the dose per infusion reduced, and if it is too high it may be increased to 7 nights per week. When iron overload is severe, and cardiac dysfunction has developed, deferoxamine should be administered by continuous intravenous infusion (Davis & Porter, 2000). There are some data suggesting that increasing the dose beyond 50–60 mg/kg in a 24-h period does not have any additional benefit. Monitoring for toxicity of deferoxamine includes regular ophthalmic (for visual fields) and audiological (for high frequency hearing loss) examinations.

Two oral chelators are currently in use. Deferiprone (L1, Apopharma, not licensed in the USA) (Hoffbrand *et al*, 2003) is a bidentate oral iron chelator, which at higher doses has been shown in non-randomized studies to increase the cardiac T2* more than deferoxamine (Davis & Porter, 2000; Anderson *et al*, 2001). The clinical significance of this is not clear. Serious toxicity includes arthritis, sometimes severe, and neutropenia. In particular, the recent documentation of mortality due to agranulocytosis in patients treated with Deferiprone has resulted in the recommendation to avoid Deferiprone in patients with DBA (Hoffbrand *et al*, 1989; Henter & Karlén, 2007). Deferasirox (ICL670, Exjade®, Novartis) is a tridentate oral iron chelator whose long half-life permits once daily dosing. Phase II and III trials in transfusion-dependent patients with a variety of underlying conditions (Nisbet-Brown *et al*, 2003) have demonstrated that doses of 20 and 30 mg/kg per day stabilize or decrease liver iron. Toxicity is mild and usually transient and generally includes rash, nausea and abdominal discomfort, elevations in creatinine, rare proteinuria and transaminase elevations. Exjade® is now approved in the United States as first line therapy for transfusional iron overload in patients two years and older. The effect of deferasirox on cardiac iron remains to be studied.

## Hematopoietic stem cell transplantation

Hematopoietic stem cell transplantation is curative in DBA (August *et al*, 1976; Iriondo *et al*, 1984; Mugishima *et al*, 1995; Willig *et al*, 1999a; Vlachos *et al*, 2001b). However, its role is controversial. The Italian and German DBA registries reported nine of 11 patients and 20 of 22 patients, respectively, who were transplanted successfully (unpublished observations). The largest series of HSCT comes from the DBAR (Lipton *et al*, 2006) where the major indication for HSCT was transfusion dependence. Two patients were also transplanted for severe aplastic anaemia and one for significant thrombocytopenia. Bone marrow was the most common source of stem cells, however four unrelated cord blood and one unrelated peripheral stem cell transplants were performed. Only one of these five DBA patients is alive. These deaths were due to toxicity and none were attributable to graft rejection or loss. The survival for allogeneic sibling *versus* alternative donor transplant was 72·7% vs. 17·1% at greater than 5 years from SCT (*P* = 0·012). Importantly the survival for patients less than 10 years of age transplanted using HLA-matched allogeneic siblings was 92·3%. More recently, successful related umbilical cord blood and unrelated donor transplants have been reported to the DBAR but the data are too early for meaningful analysis.

In general, patients with DBA, whether steroid-responsive or transfusion-dependent, may be considered for transplant prior to age 10 years, and preferably between the ages of 2 and 5 years, if an HLA-matched related donor is available. This is particularly true for transfusion-dependent patients, in whom iron overload, as described in the thalassemia transplant experience, is a significant adverse prognostic factor (Lucarelli *et al*, 1990, 2002). The actuarial likelihood of treatment independence of around 20% needs be taken into account when considering HSCT. Also, as most patients taken to HSCT are steroid non-responders, the effect of a second steroid trial can be tested prior to HSCT. Data on the conditioning regimen in DBA are relatively limited; however patients not receiving total body irradiation have a better outcome (Vlachos *et al*, 2001b). There are insufficient data supporting a recommendation for non-myeloablative/reduced intensity transplant conditioning in DBA (Gomez-Almaguer *et al*, 2003). In addition, the role of any conditioning regimen in the development of malignancy must be taken into account. One patient developed osteogenic sarcoma post-HSCT (Lipton *et al*, 2001). If possible, it is recommended that umbilical cord blood be harvested as a source of stem cells from subsequent pregnancies once a DBA-affected proband is identified. Strict criteria must be applied to ensure that the potential donor is unaffected.

While recent data on unrelated HSCT may be encouraging, the indications for HSCT for a patient without a family matched donor are limited. There are too few transplants to recommend routine unrelated donor SCT. At present, the indications for unrelated HSCT are the onset of bi- or trilineage cytopenia and/or the evolution to myelodysplasia or AML. Transfusion complications, such as occurence of red cell allosensitzation or iron chelator intolerance, may be indicators for proceeding to unrelated HSCT and should be evaluated on a case-by-case basis.

## Alternative therapies

Other treatments, summarized in Table VI, have been used in DBA over the last 30 years. These drugs appear to be largely ineffective and there is currently no evidence that any of these has a major role in the management of DBA (Geller *et al*, 1975; Dunbar *et al*, 1991; Fiorillo *et al*, 1991; Niemeyer *et al*, 1991; Gomez-Almaguer & Gonzalez-Llano, 1992;Sumimoto *et al*, 1992; Bejaoui *et al*, 1993; Gillio *et al*, 1993a, 1993b;Bastion *et al*, 1994; Brown *et al*, 1994; Olivieri *et al*, 1994; Ozsoylu, 1994; Ball *et al*, 1995; Bernini *et al*, 1995; Buchanan, 2001; Alter, 2003**).** Specific patients may respond to cyclosporine **(**Totterman *et al*, 1984; Williams *et al*, 1987; Seip & Zanussi, 1988; Leonard *et al*, 1989; Splain & Berman, 1992; Monteserin *et al*, 1993; Alessandri *et al*, 2000; Bobey *et al*, 2003) or metoclopramide (Abkowitz *et al*, 2002; Akiyama *et al*, 2005; Leblanc *et al*, 2007), but currently these responders cannot be identified *a priori*. There have been two reports on the use of valproic acid (Jabr *et al*, 2004; Jabr & Taher, 2006) and leucine (Pospisilova *et al*, 2007) with good outcomes, however these are case reports and warrant further study before any conclusions can be drawn. The known rate of remission should be considered when evaluating these small trials and anecdotes.

**Table VI:** Summary of alternative treatments*.

Treatments	Number of patients	Response	References
Androgens	>100	20%	Gomez-Almaguer and Gonzalez-Llano (1992)
High dose corticosteroids	12	7	(Ozsoylu, 1994)
	8	3 complete, 1 partial	(Bernini *et al*, 1995)
	9	5 all transient	(Buchanan, 2001)
Erythropoietin	10	1 transient	(Fiorillo *et al*, 1991; Niemeyer *et al*, 1991)
Interleukin-3	100	10%	(Dunbar *et al*, 1991; Gillio *et al*, 1993a, 1993b; Bastion *et al*, 1994; Olivieri *et al*, 1994;Ball *et al*, 1995)
Cyclosporine ± prednisone	20 (with steroids) 10 (CSA alone)	50% all transient 2 sustained response	(Totterman *et al*, 1984; Williams *et al*, 1987;Seip & Zanussi, 1988; Leonard *et al*, 1989;Splain & Berman, 1992; Monteserin *et al*, 1993;Alessandri *et al*, 2000; Bobey *et al*, 2003)
Metoclopromide	9	1 complete 2 partial on steroid taper	(Abkowitz *et al*, 2002; Akiyama *et al*, 2005;Leblanc *et al*, 2007)
	1	1 complete	
	33	2 partial	
Valproic acid	1	1 complete	(Jabr & Taher, 2006)
Leucine	1	1 complete	(Pospisilova *et al*, 2007)
Other (6-MP, cyclophosphamide,Vincristine, stem cell factor,PIXY 321, IVIG)		Largely ineffective	(Geller *et al*, 1975; Sumimoto *et al*, 1992;Bejaoui *et al*, 1993; Brown *et al*, 1994)and anectodal communications

*Data taken from Alter (2003).

ATG, antithymocyte globulin; PIXY 321, a GM-CSF/IL-3 fusion protein; IVIG, intravenous immunoglobulin; 6-MP, 6-mercaptopurine.

## Gene therapy

Gene therapy is under development for rps19 deficient DBA. Currently, it has been shown that enforced expression of rps19 improves erythroid development in primary cells from patients *in vitro* (Flygare *et al*, 2005). Model systems with rps19 deficiency in primary human hematopoietic cells have been generated (Hamaguchi *et al*, 2003). Animal models with rps19 deficiency have to be created (Matsson *et al*, 2004) to test therapeutic efficacy of the viral vectors. The roadmap to develop a human gene therapy protocol is expected to take at least 5 years.

## Outcomes

Overall, in most national registries, approximately 40% of patients with DBA are transfusion-dependent, having failed to respond or having become refractory to steroids, while 40% are steroid-dependent, and 20% are transfusion-independent on no medication (“in remission”) (Willig *et al*, 1999a; Lipton *et al*, 2006). Remission occurs in some patients who are initially steroid responsive when steroids can be stopped completely with continued maintenance of adequate hemoglobin levels. A small number of steroid non-responders may also enter remission even after prolonged transfusion dependence. The DBAR defines remission as a stable, physiologically acceptable hemoglobin, maintained for at least six months independent of corticosteroids, transfusions or other therapy. Seventy percent of the remitters in the DBAR did so within the first decade of life. Most patients have a sustained remission. However hormonal stress in pregnancy appears to be an important factor contributing to “relapse”; this may be transient.

An analysis of the North American DBAR data (Lipton *et al*, 2006) revealed 70% of the deaths were treatment-related: infections, complications of iron overload, vascular access device complication, and HSCT complications. Thirty percent of deaths are disease-related: severe aplastic anaemia and malignancy. The overall actuarial survival at greater than 40 years of age is 75·1%; 86·7% for corticosteroid-maintainable patients and 57·2% for transfusion-dependent patients. Of note, the majority of deaths in the transfusion-dependent group are a consequence of HSCT-related complications. The French registry reported a 17-month-old boy on long-term steroid therapy that died after presenting with silent peritonitis (Willig *et al*, 1999a). *Pneumocystis jiroveci* (formerly *carinii*) pneumonia has also been a notable complication in DBA patients treated with very high dose steroids (Huh *et al*, 2002). These data strongly support recommendations of judicious steroid use, adherence to chelation regimens, and the critical evaluation of transplant indications on a case-by-case basis.

Five DBAR patients have been diagnosed with severe aplastic anaemia, which presented while the patient was receiving treatment for DBA. This is consistent with the finding of decreased marrow cellularity with age in patients with DBA (Giri *et al*, 2000). Anecdotally these patients have failed medical management and have required stem cell transplantation (DBAR, unpublished observations).

## Management of adult patients

With advances in supportive care, transfusion and steroid therapy, and stem cell transplantation, DBA is evolving from an exclusively childhood disorder to a disorder also affecting adults. There are known DBA patients who survive late into adulthood, as well as patients newly diagnosed as adults. Heterogeneity of presentation and atypical clinical features may be confounding factors in the diagnosis of DBA. Important causes of acquired pure red cell aplasia are enumerated in Table I. The diagnosis of DBA may have therapeutic implications, as some individuals who are first recognized in adulthood may respond to steroid therapy (Balaban *et al*, 1985). Due to a paucity of data regarding adult patients there are a number of unanswered questions about natural disease progression and what factors lead to long-term survival. It is unclear whether DBA worsens over time, however, age-related decreases in erythroid and granulocyte/macrophage progenitor numbers have been reported (Casadevall *et al*, 1994). Cancer is also part of the natural history but the cancer risk, including the incidence of AML and myelodysplastic syndrome (MDS), is unknown in adults. Also, DBA may need to be considered in the differential diagnosis of patients who present with aplastic anaemia.

There are also a number of considerations that may be intrinsic to adult patients, namely postadolescence and hormonal changes; aging; concomitant chronic diseases; sexually transmitted diseases; lifestyle diseases; fertility and pregnancy (Table VII); psychosocial and financial concerns. Thus, the lifetime management of DBA patients should be by a multidisciplinary team involving pediatric and adult hematologists, internists and subspecialists, as needed. Physicians should be aware of the possibility of developing aplastic anaemia, AML, MDS, or solid tumors. In general, asymptomatic adults with known DBA mutations should be followed like children in remission.

**Table VII:** Complications of pregnancy in women with Diamond Blackfan anaemia (DBA).

Maternal
Increased anaemia with a loss of steroid responsiveness or an increase in transfusion requirement
Accelerated haemochromatosis during respite from chelation
Risk of:
Abortion
Preterm delivery
Pre-eclampsia
Caesarean section
Placental vasculopathy
Fetal
Fetal death
Intra-uterine growth retardation
Prematurity
Recurrence of DBA including severe form, e.g. profound neonatal anaemia and hydrops fetalis
Risk of congenital abnormalities

Data from Faivre *et al* (2006).

## Management of pregnancy

Improvements in the management of pregnant women with DBA have resulted in an increase in survival, quality of life and reproductive potential. Consequently, many affected women who have reached childbearing age in good condition are now willing and able to experience pregnancy. The recurrence risk in offspring has been discussed and follows that of an autosomal dominant disorder.

No prospective study on pregnancy in women with DBA is available. Nevertheless a number of reported cases in the literature tend to highlight the risks associated with pregnancy (Alter *et al*, 1999). Complications of pregnancy include complications in the mother, the child, or, both. A recent study, relying on the French and German registries, reports on 60 pregnancies in 44 women, and emphasizes the potential hazards, including fetal loss, pre-eclampsia, preterm delivery, intra-uterine death, infants with intra-uterine growth retardation and children with congenital abnormalities (Faivre *et al*, 2006) (Table VII). The etiology of such complications remains a matter of debate and may be either a direct result of the DBA mutation, chronic anaemia, iron overload, DBA treatment, or some combination of any, or all, of these. Of interest, some of the observed complications are similar to those reported in placental vascular disease. Although a placental vasculopathy has not been documented in pregnant women with DBA, treatment with aspirin has been beneficial in some cases. There is no significant correlation between the outcome of pregnancy and fetal DBA status.

The additional iron burden as a consequence of a new transfusion requirement should be dealt with postpartum as chelation is contraindicated during pregnancy (Skordis *et al*, 1998; Aessopos *et al*, 1999). Deferoxamine is known to be teratogenic at high doses in rodents while deferasirox is not. However, neither medication is licensed for use in pregnancy despite reports of safe administration of deferoxamine in a limited number of pregnant thalassemia patients and pregnant women with iron poisoning (Singer & Vichinsky, 1999). Chelation should cease when the patient is planning to become pregnant, or as soon as a pregnancy is recognized.

To the extent possible, pregnancies should be planned. When indicated, an intensification of iron chelation should be performed before the onset of pregnancy. Any woman with DBA contemplating pregnancy should undergo a thorough evaluation of any feature that may interfere with pregnancy outcome, including the presence of blood-borne infections, iron overload and related diabetes mellitus, hypothyroidism or cardiomyopathy. Some of these will have to be regularly assessed during the pregnancy thus care should be administered in a high-risk obstetrical practice in collaboration with hematologists and other appropriate subspecialists. Intensified ultrasound and Doppler follow-up are recommended to screen for congenital abnormalities and hydrops fetalis in the fetus, and to assess a potential placental vascular disorder. In cases where the father of the child is the DBA-affected individual, the pregnancy should also be considered high risk and closely monitored for signs of fetal distress, hydrops fetalis and other complications of the fetus (Rogers *et al*, 1997).

The hemoglobin concentration to be maintained in pregnancy has not been established. The available literature on anaemia and pregnancy outcome is mainly devoted to severe iron deficiency and sickle cell disease and is not relevant in this setting. In thalassemia patients, the usually recommended hemoglobin in pregnancy is 100 g/l (Skordis *et al*, 1998; Aessopos *et al*, 1999). Chronically transfused DBA patients should be maintained at that hemoglobin level. Steroid responsive women often experience an increased steroid requirement or become transfusion-dependent during pregnancy. In most cases, an increase in the prepregnancy steroid dose fails to maintain a satisfactory hemoglobin level. It is also unlikely that steroid treatment initiation in a previously untreated woman will be efficacious (Alter *et al*, 1999; Faivre *et al*, 2006). Ultimately, steroid toxicity must be considered in both the mother and the fetus. Transfusion therapy is therefore usually necessary.

In conclusion, much of the new data suggesting management pathways derive from the analysis of data from worldwide DBA registries. Relevant supporting literature is cited, when available, but clinical recommendations are largely based on the experience of veteran clinicians (Appendix I) and extrapolation from similar clinical situations. When consultation is recommended, it should be with specialists who have experience in the management of patients with IBMFS. Families and patients should be referred to DBA support groups in their area (http://www.dbafoundation.org).

## Appendix I

Participants in the Sixth Annual Diamond Blackfan Anemia International Consensus Conference, New York, April 16–18, 2005 – in alphabetical order:

Blanche P. Alter, MD, MPH, National Cancer Institute, Bethesda, MD, USA

Eva Atsidaftos, MA, Schneider Children's Hospital, Albert Einstein College of Medicine, New Hyde Park, NY, USA

Sarah Ball, MD, St. George's Hospital Medical School, London, UK

Monica Bessler, MD, PhD, Washington University School of Medicine, St. Louis, MO, USA

Niklas Dahl, MD, PhD, Uppsala University, Uppsala, Sweden

Irma Dianzani, MD, PhD, Università del Piemonte Orientale, Novara, Italy

Yigal Dror, MD, The Hospital for Sick Children, Toronto, Ontario, Canada

Steven Ellis, PhD, University of Louisville, Louisville, KY, USA

Hanna Gazda, MD, Dana-Farber Cancer Institute, Boston, MA, USA

Bertil Glader, MD, PhD, Stanford University, Palo Alto, CA, USA

Karen Gripp, MD, DuPont Hospital, Wilmington, DE, USA

Elizabeth M. Kang, MD, National Institute of Allergy and Infectious Diseases, National Institutes of Health, Bethesda, MD, USA

Stefan Karlsson, MD, PhD, Lund University, Lund, Sweden

Thierry Leblanc, MD, Hôpital Saint-Louis, Paris, France

Jeffrey M. Lipton, MD, PhD, Schneider Children's Hospital, Albert Einstein College of Medicine, New Hyde Park, NY, USA

Johnson M. Liu, MD, Schneider Children's Hospital, Albert Einstein College of Medicine, New Hyde Park, NY, USA

Fabrizio Loreni, PhD, Università Tor Vergata, Roma, Italy

Joerg Meerpohl, MD, Zentrum für Kinderheilkunde und Jugendmedizin, Universitaetsklinikum Freiburg, Freiburg, Germany

Carole Paley, MD, Novartis, East Hanover, NJ, USA

Charles Peterson, MD, National Heart, Lung, and Blood Institute, Bethesda, MD, USA

Pankaj Qasba, PhD, National Heart, Lung, and Blood Institute, Bethesda, MD, USA

Ugo Ramenghi, MD, Università di Torino, Torino, Italy

Sujit Sheth, MD, MS, Columbia University, New York, NY, USA

Akiko Shimamura, MD, PhD, Children's Hospital, Boston, MA, USA

Colin Sieff, MD, Dana-Farber Cancer Institute, Boston, MA, USA

Adrianna Vlachos, MD, Schneider Children's Hospital, Albert Einstein College of Medicine, New Hyde Park, NY, USA

Winfred Wang, MD, St. Jude Children's Hospital, Memphis, TN, USA
